# Connexin Lateralization Contributes to Male Susceptibility to Atrial Fibrillation

**DOI:** 10.3390/ijms231810696

**Published:** 2022-09-14

**Authors:** Simon Thibault, Anh-Tuan Ton, François Huynh, Céline Fiset

**Affiliations:** 1 Research Center, Montreal Heart Institute, Montreal, QC H1T 1C8, Canada; 2 Faculty of Pharmacy, Université de Montreal, Montreal, QC H3T 1J4, Canada

**Keywords:** atrial fibrillation, sex differences, connexins, conduction, orchiectomy, sex hormones

## Abstract

Men have a higher risk of developing atrial fibrillation (AF) than women, though the reason for this is unknown. Here, we compared atrial electrical and structural properties in male and female mice and explored the contribution of sex hormones. Cellular electrophysiological studies revealed that action potential configuration, Na^+^ and K^+^ currents were similar in atrial myocytes from male and female mice (4–5 months). Immunofluorescence showed that male atrial myocytes had more lateralization of connexins 40 (63 ± 4%) and 43 (66 ± 4%) than females (Cx40: 45 ± 4%, *p* = 0.006; Cx43: 44 ± 4%, *p* = 0.002), with no difference in mRNA expression. Atrial mass was significantly higher in males. Atrial myocyte dimensions were also larger in males. Atrial fibrosis was low and similar between sexes. Orchiectomy (ORC) abolished sex differences in AF susceptibility (M: 65%; ORC: 38%, *p* = 0.050) by reducing connexin lateralization and myocyte dimensions. Ovariectomy (OVX) did not influence AF susceptibility (F: 42%; OVX: 33%). This study shows that prior to the development of age-related remodeling, male mice have more connexin lateralization and larger atria and atrial myocyte than females. Orchiectomy reduced AF susceptibility in males by decreasing connexin lateralization and atrial myocyte size, supporting a role for androgens. These sex differences in AF substrates may contribute to male predisposition to AF.

## 1. Introduction

Atrial fibrillation (AF) is the most common type of sustained cardiac arrhythmia, affecting between 1–2% of the population [1,2,3]. Male sex is one of its most important risk factors, and is associated with a 1.5–2 times greater risk than female sex [1,4,5,6,7,8,9]. Furthermore, the prevalence of AF is higher in men than in women in all age groups [10]. The clinical presentation of AF also differs between sexes, with men having not only a higher prevalence but also an earlier onset, while AF in women tends to be more severe, and is more often associated with a history of stroke and atrial fibrosis [5,11,12,13,14]. It thus appears that men are more likely to develop AF, and require less adverse remodeling than women, strongly suggesting sex differences in the pathogenesis of AF.

Although the mechanisms leading to AF are diverse, they require the interaction between trigger and maintenance AF substrates [1,2,15,16,17,18]. First, triggers act as the initiators of arrhythmia. These are often caused by abnormal automaticity or ectopic firing. Then, AF can only be maintained if the electrophysiological and structural properties of the atria are favorable. This can occur in the presence of electrophysiological remodeling, which includes changes in ionic currents. Alterations in K^+^ and Ca^2+^ currents will alter action potential duration (APD) and effective refractory period (ERP), and thus cell excitability. Generally, a shortening of APD is thought to promote re-entry, and is considered a hallmark in the mechanisms by which AF begets AF [2,15,19]. On the other hand, changes in Na^+^ currents (I_Na_) instead affect cell depolarization and conduction. Electrical conduction is also highly dependent on connexins, as they facilitate the cell-to-cell conduction by allowing the electrochemical coupling of adjacent cells at the intercalated disks. The atria expressed both connexin 40 (Cx40) and connexin 43 (Cx43) [20,21,22]. Remodeling of the connexins is commonly reported in patients with AF. Notably, their reduced expression and increased lateralization can slow the conduction velocity [20,21,22,23]. In addition, lateralization also increases conduction heterogeneity by promoting non-linear propagation of electrical impulses through the atria. Connexin remodeling creates additional substrates to maintain AF [20]. AF is also associated with structural remodeling, characterized by the development of atrial fibrosis and an increase in atrial size. This structural remodeling also slows the conduction velocity through the atria, and thus plays a key role in the maintenance of AF [15].

Considerable progress has been made over the past decades in understanding the complex pathophysiology of AF [2]. Notably, it has become clear that electrical and structural remodeling of the atria are critical processes involved in the maintenance of AF. However, very little is known about potential sex differences in these mechanisms despite the clear sex difference in AF prevalence [7,24,25]. Therefore, we realized the present study to determine whether there are sex differences in maintenance AF substrates that might contribute to the male prevalence of AF. Specifically, we compared the electrical and structural properties of atrial myocytes in healthy adult male and female mice, prior to the onset of age-related remodeling to avoid potential confounding factors.

Mechanisms underlying sex differences in ventricular electrophysiology have been elucidated using animal models, including mice. Among others, previous studies by our group have shown that, as seen in humans, ventricular repolarization and QTc interval are shorter in CD−1 male mice compared to their female counterparts, due to androgen-dependent upregulation of repolarizing K^+^ channels [26,27,28]. Based on these observations, we chose as the experimental model for this study the CD−1 mice.

## 2. Results

AF maintenance mechanisms are complex, and involve electrical and structural remodeling characterized by decreased conduction velocity and changes in atrial action potential (AP). Alterations in several ionic currents have been associated with changes in APD that promote AF. Along with the development of interstitial fibrosis and structural remodeling, a reduction in I_Na_ density and/or a change in the expression and/or distribution of connexins may contribute to slowing the conduction velocity in the atria. Therefore, in this study to investigate potential sex differences in the mechanisms involved in the maintenance of AF, we examined these parameters.

### 2.1. There Are No Sex Differences in AP Configuration and Ionic Currents

**Figure 1:**
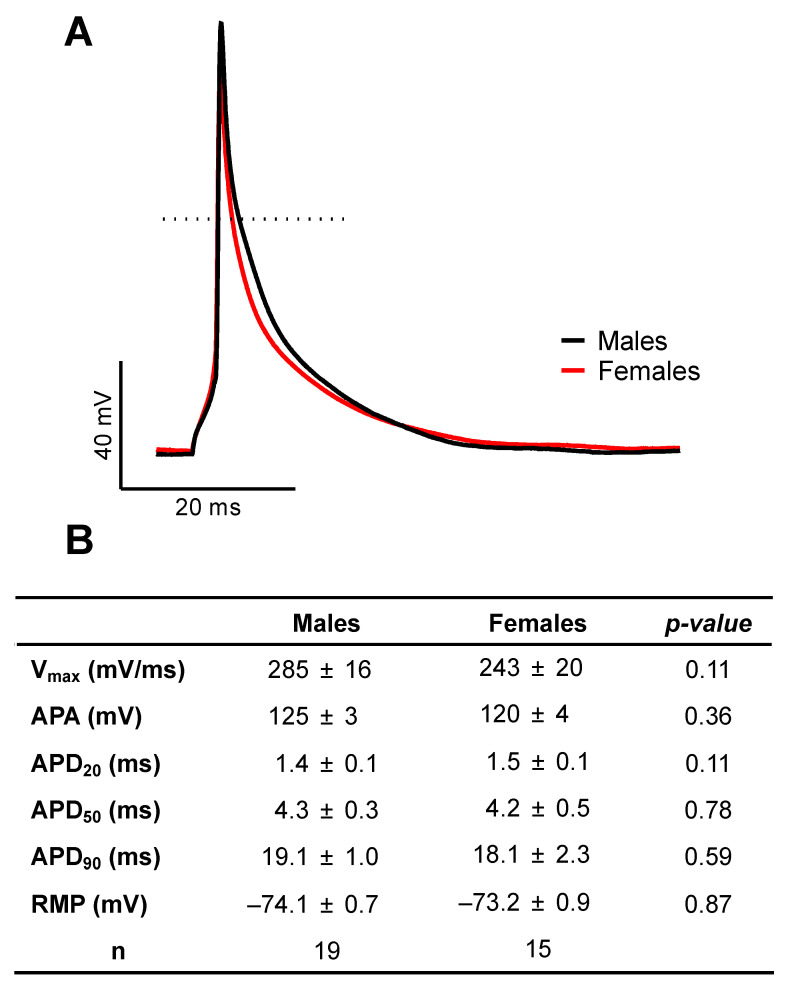
**Similar action potential configuration in left atrial myocytes of male and female mice.** (**A**)**.** Representative recordings of male (black) and female (red) action potentials recorded using perforated patch-clamp techniques at 4 Hz at 37 °C. Dotted line displays 0 mV. (**B**)**.** Summary table of mean values of action potential parameters: maximum upstroke velocity (Vmax), action potential amplitude (APA), action potential duration at 20, 50 and 90% of repolarization (APD_20_, APD_50_ or APD_90_) and resting membrane potential (RMP). No statistical difference was observed between males and females.

**Figure 2:**
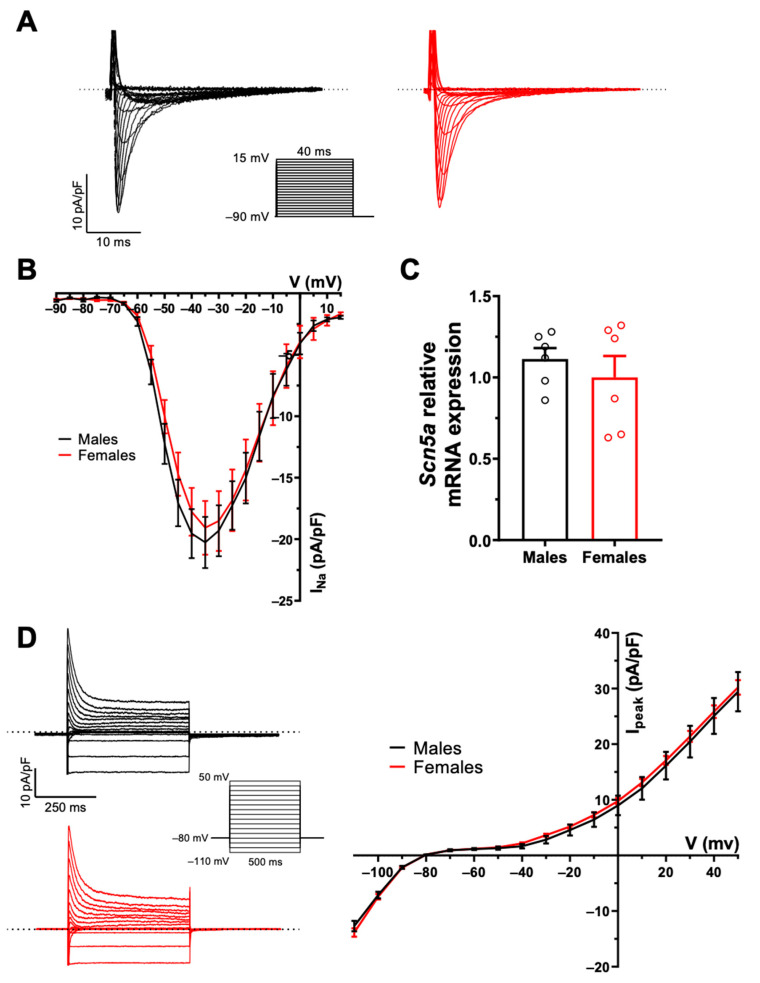
**No sex difference in Na^+^ and K^+^ currents in mouse left atrial myocytes.** (**A**). Typical recordings of I_Na_ from male (black) and female (red) left atrial myocytes. The inset shows voltage protocol here and in panel D. (**B**). Mean IV curves of I_Na_ show comparable current density between male and female left atrial myocytes (at −35 mV: M: −20.3 ± 2.0 pA/pF, n = 11, N = 3; F: −19.1 ± 2.1 pA/pF, n = 13, N = 2; *p* = 0.70). (**C**). mRNA expression of the *Scna5* gene, underlying the Na_V_1.5 channel, was not different between male and female left atrial tissues (M: 1.11 ± 0.07, n = 6; F: 1.00 ± 0.13, n = 6; *p* = 0.46). (**D**, **left**). Typical recordings of the total K^+^ current (I_peak_) from male (black) and female (red) left atrial myocytes. (**D**, **Right**). Mean IV curves of I_peak_ show no sex difference in left atrial myocytes (at +30 mV: M: 20.5 ± 2.8 pA/pF, n = 13, N = 5; F: 21.4 ± 1.0 pA/pF, n = 26, N = 9, *p* = 0.78). Welch’s t-test was used due to unequal variances (*p* > 0.05 at all voltages). Dotted lines represent the zero current.

AP configuration and ionic currents are key determinants of atrial electrical activity. Accordingly, in the first series of experiments, we compared atrial AP and major ionic currents between male and female mice to determine whether differences in cellular electrophysiological properties could contribute to the male AF prevalence. Figure 1A shows typical examples of AP recorded from freshly isolated left atrial myocytes obtained from a male and a female mouse, and illustrates that AP morphology was similar between the two groups. Detailed analysis of the AP parameters summarized in Figure 1B indicates that AP maximum upstroke velocity (Vmax), AP amplitude (APA), APD, and resting membrane potential were all similar between the two sexes. Consistent with the AP data we observed no differences in Na^+^ current (I_Na_) and total K^+^ current (I_peak_) in atrial myocytes of male and female mice. Indeed, Figure 2A,B show that I_Na_ is similar in both groups (at −35 mV: M: −20.3 ± 2.0 pA/pF, n = 11, N = 3; F: −19.1 ± 2.1 pA/pF, n = 13, N = 2; *p* = 0.70). Moreover, there was no differences in the transcript levels of *Scn5a*, the underlying α-subunit Na^+^ channel between male and females (Figure 2C). In addition, the density of the total K+ current was also identical between males (at +30 mV: 20.5 ± 2.8 pA/pF, n = 13, N = 5) and female (21.4 ± 1.0 pA/pF, n = 26, N = 9, *p* = 0.78) atrial myocytes (Figure 2D). Collectively, these cellular electrophysiological results indicate that differences in AP configuration, depolarizing Na^+^ and repolarizing K^+^ currents do not contribute to the male predisposition to AF.

### 2.2. Sex Differences in Connexin Lateralization

**Figure 3:**
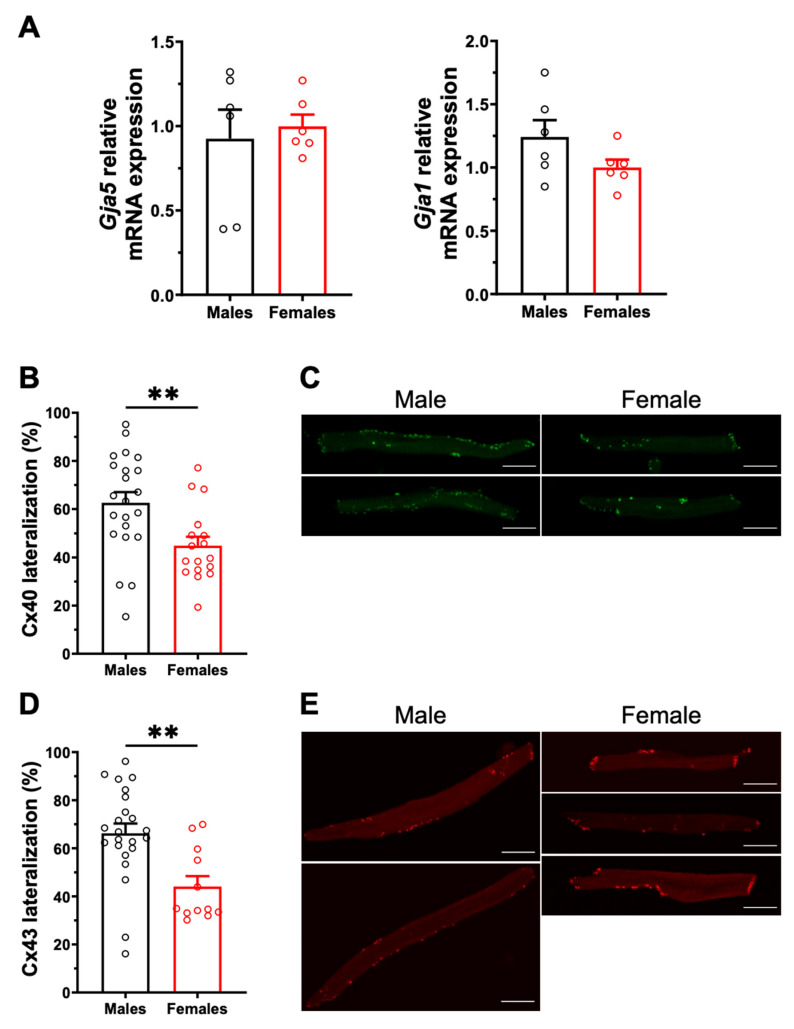
**Gene expression and cellular distribution of Cx40 and Cx43.** (**A**). No sex differences were observed in mRNA expression of *Gja5* (left) and *Gja1* (right) encoding for Cx40 and Cx43 (n = 6/group), respectively. (**B**). The percentage of Cx40 lateralization, defined as the percentage of positive signal present at the lateral membrane, is higher in male left atrial myocytes compared to females (M: 63 ± 4%, n = 22, N = 4; F: 45 ± 4%, n = 17, N = 3; ** *p* = 0.005). (**C**). Representative example of the Cx40 distribution (in green) in male and female atrial myocytes. (**D**). Scatter plot of Cx43 lateralization in male and female atrial myocytes (M: 66 ± 4%, n = 23, N = 4; F: 44 ± 4%, n = 12, N = 3; ** *p* = 0.002). (**E**). Representative examples of Cx43 distribution (in red). All scale bars represent 20 µm. ** *p* < 0.01.

Cx40 and Cx43 are also major contributors to atrial conduction as they provide electrochemical coupling to adjacent cells. We therefore compared their expression and distribution patterns in male and female mouse atrial myocytes. qPCR analysis showed that mRNA expression of *Gja5* and *Gja1*, encoding Cx40 and Cx43 respectively, was comparable in male and female atrial tissues, as shown in Figure 3A. However, immunofluorescence experiments performed on isolated atrial myocytes showed a more pronounced lateralization of the cellular distribution of Cx40 in males (M: 63 ± 4%, n = 22) than in females (45 ± 4%, n = 17, *p* = 0.006). Similarly, a significantly higher proportion of Cx43 was expressed at the lateral membranes of male myocytes (M: 66 ± 4%, n = 23) compared to females (F: 44 ± 4%, n = 12; *p* = 0.002) (Figure 3B–E). These results suggest that the greater lateralization of Cx40 and Cx43 in male atrial myocytes may promote non-linear conduction, and thus contribute to male vulnerability to AF.

### 2.3. Potential AF Anatomical Substrates

#### 2.3.1. No Sex Differences in Atrial Interstitial Fibrosis and Fibrosis Markers

**Figure 4:**
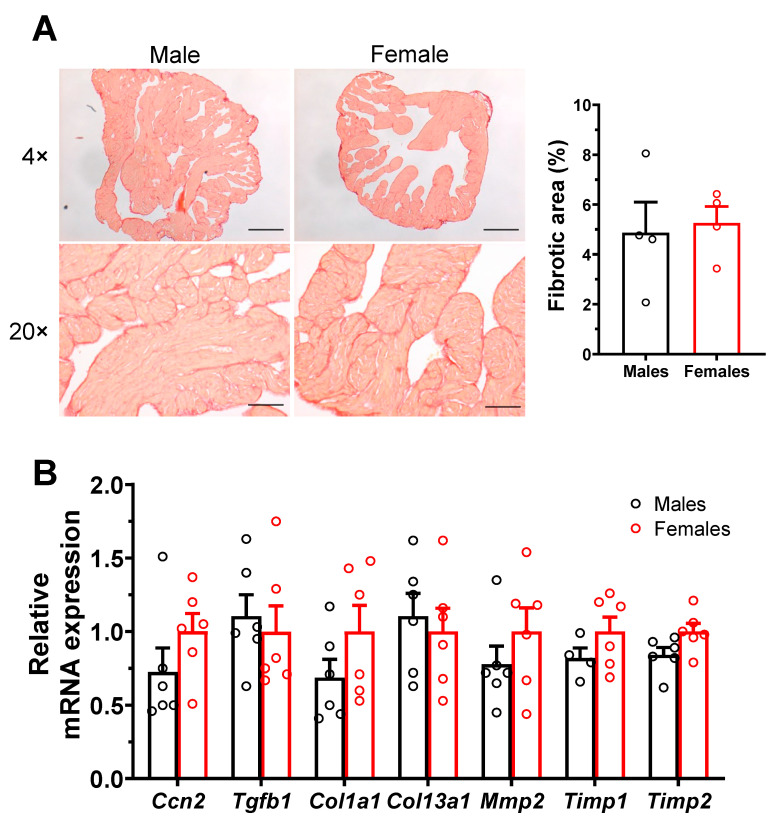
**Similar level of interstitial fibrosis in male and female atrial myocytes.** (**A**). Typical collagen picrosirius red staining (stained red) of left atria of male and female mice, at a 4× and 20× magnification zoom. Scale bars represent 100 and 500 µm, at 4× and 20×, respectively. The scatter plots reveal no differences in atrial fibrous content between males and females (M: 4.9 ± 1.2%, n = 4; F: 5.3 ± 0.7%, n = 4; *p* = 0.79). (**B**). qPCR analysis shows that the mRNA expression level of genes involved in fibrosis pathways was similar between male and female atria (n = 4–6/group).

As interstitial fibrosis is known to negatively impact atrial electrical conduction and promote AF, we compared the amount of atrial interstitial fibrosis in male and female mice. The left atrial collagen content assessed using picrosirius red staining was found to be low and comparable in both groups (M: 4.9 ± 1.2%, n = 4; F: 5.3 ± 0.7%, n = 4; *p* = 0.79) (Figure 4A). In addition, qPCR experiments were also performed on left atrial tissues, and no sex differences were observed in mRNA expression of various genes involved in fibrosis pathways (Figure 4B). 

#### 2.3.2. Sex Differences in Atrial Structural Properties

**Figure 5:**
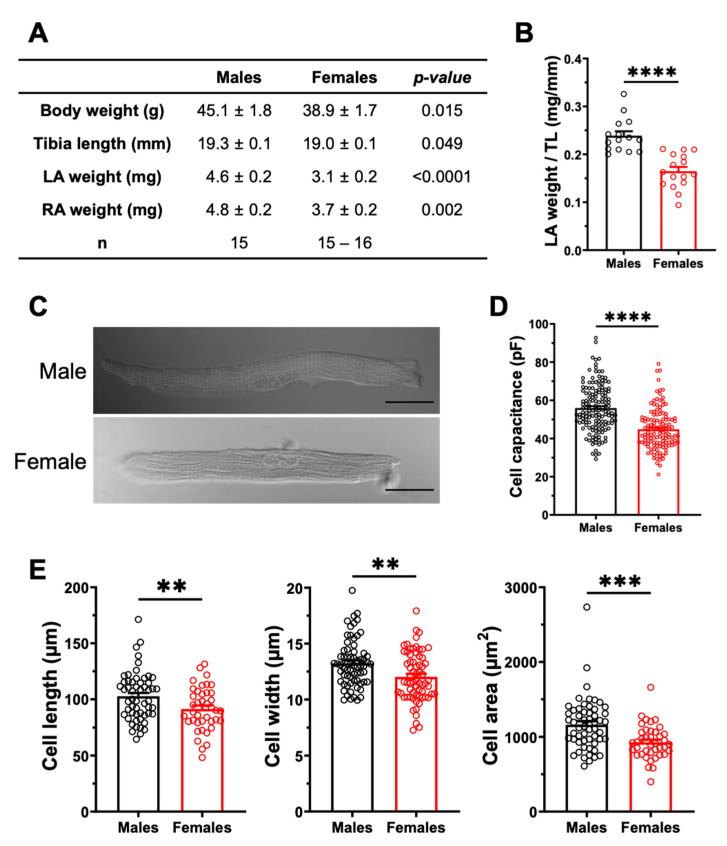
**The left atria are heavier, and the atrial myocyte capacitances are greater in males.** (**A**). Summary table of the numerical values of morphometric measurements of male and female mice. (**B**). The scatter plot displaying left atria weight normalized to tibia length (TL) shows a higher ratio in male than in female mice (M: 0.24 ± 0.01 mm/mg, n = 15; F: 0.16 ± 0.01 mm/mg, n = 16; **** *p* < 0.0001). (**C**). Representative examples of atrial myocytes isolated from male and female mice obtained from connexin immunofluorescence experiments, viewed under brightfield confocal microscopy. Scale bars represent 20 µm. (**D**). The cell capacitance of left atrial myocytes are higher in males than in females (M: 55.9 ± 1.0 pF, n = 149, N = 20; F: 44.8 ± 0.9 pF, n = 129, N = 17; **** *p* < 0.0001). (**E**). The length, width and cell area of left atrial myocytes are greater in males than in females (M: n = 55–68, N = 3; F: n = 40–67, N = 68) (** *p* < 0.01, *** *p* < 0.001). Cellular parameters in E were obtained from cells used for Ca^2+^ transient experiments performed for a separate study submitted to International Journal of Molecular Sciences (IJMS) (unpublish).

We also examined the atrial structural properties, as they may affect atrial conduction velocity, and thus constitute another potential anatomical substrate for AF. The results presented in Figure 5A indicate that the left atrial weight was significantly higher in males than in females, even after normalizing to body weight or tibial length. Specifically, when normalized to tibial length, the left atrial weight was 45% (*p* < 0.0001) larger in males than in females (Figure 5B). Left atrial cell capacitance was 25% (*p* < 0.0001) higher in males than in females, as shown in Figure 5D. In keeping with these data, the cell dimensions (length, width, and surface area) of male atrial myocytes were all larger than those of females (Figure 5E). Taken together, these results suggest that in these healthy animals, males have higher atrial mass than females due to the larger size of their myocytes, which provides a substrate for AF. On the other hand, differences in fibrosis are not a contributing factor to AF.

### 2.4. Influence of Gonadectomy on AF Susceptibility

**Figure 6:**
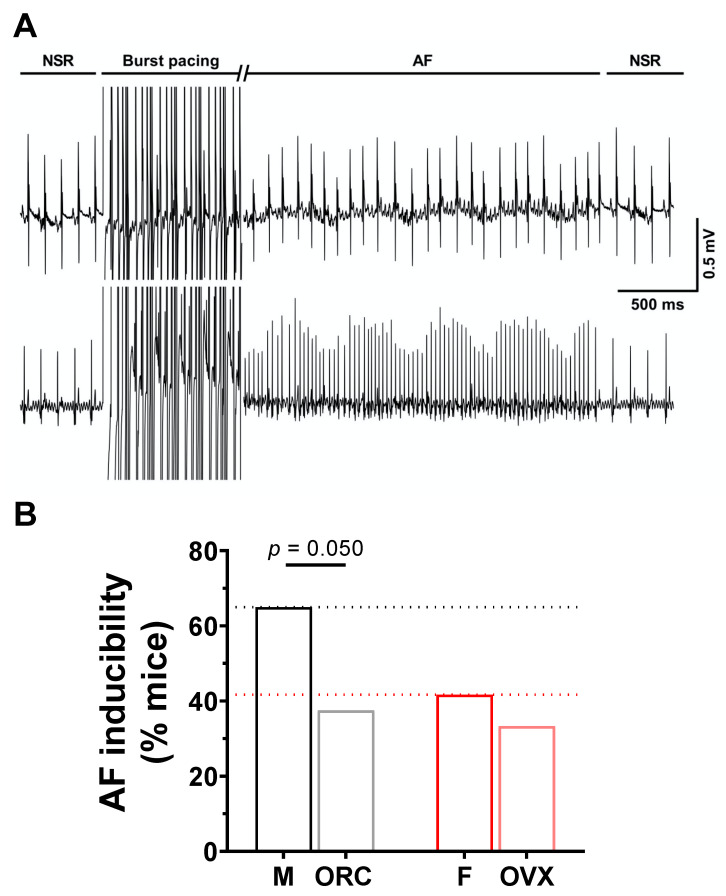
**Orchiectomy reduces susceptibility of males to AF.** (**A**). Typical recording of simultaneous surface ECG and intracardiac ECG (iECG) with induction of AF in response to burst pacing, followed by spontaneous recovery to NSR. The burst pacing duration has been cut for easier graphical illustration. (**B**). The bar graphs summarize data obtained on AF inducibility in intact and gonadectomized male and female mice in response to burst stimulation protocols (M: 65%, 13/20; ORC: 38%, 6/16; F: 42%, 10/24; OVX: 33%, 4/12). While ovariectomy has no effect on AF susceptibility in females, orchiectomy reduced AF susceptibility of males. The male and orchiectomized groups were compared using the one-sided chi square test (*p* = 0.050). Data for intact male (M) and female (F) presented in panel B represent a subset of data of a separate study submitted to International Journal of Molecular Sciences (IJMS) (unpublish).

**Figure 7:**
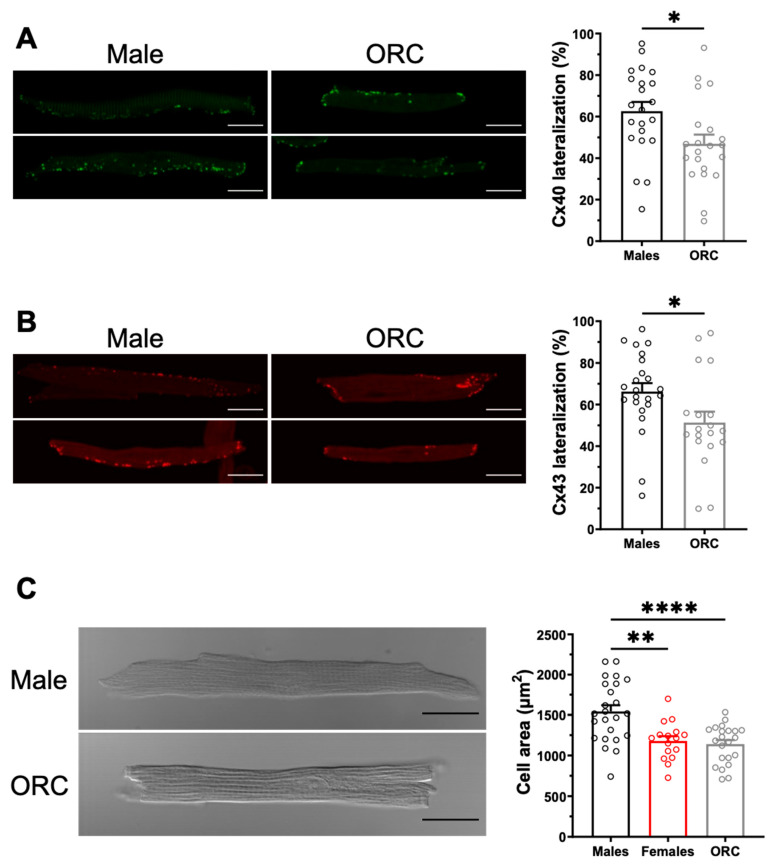
**Orchiectomy reduces connexin lateralization and cell size in males.** (**A**,**B**). **Left.** Representative examples of the distribution of Cx40 (in green, panel (**A**)) and Cx43 (in red, panel (**C**)) in male and ORC atrial myocytes. **Right.** Lateralization of Cx40 (panel (**A**)) and Cx43 (panel (**B**)) in left atrial myocytes is reduced by orchiectomy (ORC) compared to control males. (**C**). **Left.** Typical images of left atrial myocytes from male and ORC mice. **Right.** Scatter plot of cell area of male and ORC left atrial myocytes from connexin immunofluorescence experiments. Scale bars represent 20 µm. * *p* < 0.05, ** *p* < 0.01, **** *p* < 0.0001.

To explore the contribution of sex hormones in the sex-related differences reported above, we next used male orchiectomized (ORC) and female ovariectomized (OVX) mice. The EPS results (Figure 6) obtained with these animals show that although the incidence of AF was comparable between OVX and intact female mice (F: 42%, 10/14; OVX: 33%, 4/12, *p* = 0.31), orchiectomy reduced AF susceptibility in males (M: 65%, 13/20; ORC: 38%, 6/16, *p* = 0.050). These results suggest that androgens are involved in AF susceptibility in males, therefore male ORC mice were examined in more detail. As shown by the immunofluorescence data reported in Figure 7A,B, staining of Cx40 and Cx43 at the lateral membranes was significantly less apparent in ORC compared to intact males, suggesting that androgens contribute to the greater lateralization of connexins in intact males. Finally, data reported in Figure 7C shows that cell length, width, and surface area were all reduced in ORC males compared to intact males, though androgen deficiency had no significant effect on atrial mass (data not shown). In summary, atrial cell size and connexin lateralization are both affected by androgens, and are likely to help explain the lower AF susceptibility of ORC mice compared to control males. These data strongly support a contributory role of androgens in the sex difference in AF.

## 3. Discussion

The present study aimed to investigate sex differences in AF mechanisms with a focus on maintenance substrates of AF. Our results reveal that AP configuration, depolarizing Na^+^ current and repolarizing K^+^ currents do not differ between left atrial myocytes from male and female mice. However, we found that males have higher atrial mass and myocyte dimensions than females. We also identified sex differences in the cellular distribution of connexins. A higher proportion of both Cx40 and Cx43 were more lateralized in atrial myocytes from males. Orchiectomy was found to reduce AF susceptibility in response to burst pacing protocols, as well as to abolish sex differences in cell dimensions, and to reduce connexin lateralization compared to intact males. These results highlight a major role for androgens in mechanisms contributing to sex differences in AF.

Sex differences have been reported for many other types of arrhythmias besides AF [7,14]. Interestingly, sex differences in electrocardiogram (ECG) parameters were first described by Bazett in 1920 [29]. We previously established that mice exhibit sex differences in ventricular repolarization, and that androgens are responsible for these differences [26,27,28]. It is now well recognized that QTc interval and ventricular repolarization are faster in males, due to larger K^+^ currents in males of many species, including humans. This shortens APD and the refractory period in males, and explains most of the sex differences seen in ventricular arrhythmias, notably in torsades de pointes and ventricular fibrillation [9,30]. Although sex differences in ventricular electrophysiology have attracted considerable attention, knowledge in sex differences in atrial electrophysiology remains very limited. Since the ventricles and atria share most ion channels, it is often thought that repolarization may also be faster in the atria, and contribute to male susceptibility to AF [4,6,9,31,32]. However, the limited number of clinical studies comparing male-female atrial electrophysiology have reported no sex difference in the APD or AERP between men and women [33,34,35]. Consistent with these clinical observations, in mouse, we found no sex differences in atrial APD or K^+^ currents, indicating that sex differences in atrial repolarization do not contribute to male predisposition to AF. The difference between atrial and ventricular electrophysiology highlights the chamber-specific regulation of ion channels and the need to study the electrophysiological properties of atrial myocytes, rather than inferring from ventricular data to have a clear understanding of the mechanisms underlying sex differences in AF.

We found that atrial size and atrial myocytes are larger in male than in female mice. Since the difference in atrial mass (~50%) is greater than that observed in cell capacitance (~25%), this suggests that other mechanisms are also involved. For example, it is possible that the number of atrial myocytes is higher in males, which would increase the atrial mass. This could explain why cell capacitance is reduced by orchiectomy, while atrial mass is not significantly reduced. Consistent with our results, clinical data also indicate that left atrium is larger in men than in women [9,11,34]. These data support the notion that greater atrial dimension is a risk factor for AF, as it increases susceptibility to conduction disturbances, re-entries, and thus facilitates the maintenance of AF by increasing the length of the conduction pathway [34,36]. Clinical data have reported that in the presence of AF, women have more atrial fibrosis than male patients, reflecting their more severe remodeling [9,11]. However, our results indicate that there is no difference in fibrosis in healthy male and female mice when studied prior to the development of AF and age-related risk factors.

Atria are the most heterogenous cardiac tissue in terms of connexin. They contain both Cx40 and Cx43, and show variable expression within the atria. In addition, the study of connexins is technically very difficult, with few direct measurements of connexin activity [20]. The immunofluorescence results reported here show that atrial myocytes from male mice have a more pronounced lateralization of both Cx40 and Cx43. Moreover, this sex difference in connexin lateralization was abolished by orchiectomy, suggesting a role of androgens. AF have been associated with numerous perturbations of connexins, including changes in their expression, distribution, phosphorylation, and gating properties/conductance [20,21,22,23]. In patients with AF, however, decreased expression and lateralization of connexins are most commonly reported, and have been shown to slow conduction and favor non-longitudinal propagation of electrical impulse, respectively. Connexin disturbances are also known to be implicated in many diseases other than AF, including heart failure, cardiomyopathy and ischemia, as well as fibrosis and ageing [20]. With the exception of genetic mutations, it is not clear whether the connexin disturbances observed in AF patients are a cause or a consequence of their arrhythmia. Interestingly, the sex differences we observed in connexins distribution preceded AF, as they were observed in healthy young adult mice, supporting a role of connexin remodeling in the pathogenicity of AF.

In this study, we showed that orchiectomy reduced the inducibility of AF of males, while ovariectomy had no effect in females. In orchiectomized males, AF susceptibility, myocytes dimensions and connexin distribution were similar to those of females, suggesting that these parameters are regulated by androgens. These results provide new information on the contribution of sex hormones to atrial remodeling associated with AF. These findings may help to better understand the discrepancy over the relationship between androgens and AF [37]. Indeed, data on the contribution of sex hormones to the pathogenesis of AF are very conflicting. For instance, a few studies have associated low testosterone levels with a significantly increased risk of AF, while a number of studies reported the opposite, where elevated testosterone levels were associated with an increased risk of AF [38,39,40,41,42,43]. Interestingly, AF has also been reported among several cases of anabolic steroid abuse [44,45]. In addition, preclinical data is also conflicting, with some studies reporting beneficial effects of orchiectomy, while others show an increased vulnerability [46,47,48]. This apparent discrepancy is probably due to different experimental designs and conditions.

Based on clinical data, it is unclear whether female sex hormones might be involved in AF. Menopause coincides with an increase in AF incidence [4], and although there is a significant decrease in estrogens, the higher incidence of AF could also be related to changes occurring during this period in certain risk factors, such as body mass index (BMI), blood pressure and cholesterol levels [49,50]. Clinical studies related to estrogens and anti-estrogens are also conflicting, as it has been reported that the two have positive effects on AF risk in some studies, and negative ones in other studies [51,52,53,54]. The potential involvement of estrogen may not be ruled out, but from our data it appears that female sex hormones do not contribute to sex differences in AF occurring before menopause, older age, and in AF that occurs regardless of pathological conditions and other known risk factors [38,39,40,41,42,43].

## 4. Methods 

### 4.1. Animals

Male and female CD−1 mice from Charles River (St-Constant, Qc, Canada) were used at 4–5 months. The left atrium was used, as it is particularly vulnerable to AF [2,55]. All experiments were approved by the Montreal Heart Institute Animal Care Committee (2015-80-03, 2018-80-02 and 2021-80-01), and were conducted in accordance with the Canadian Council on Animal Care (CCAC) and the *Guide for the Care and Use of Laboratory Animals*, published by the National Research Council (NIH Publication No 85-23, 8th ed. 2011). 

### 4.2. Gonadectomies

Male and female mice were gonadectomized as previously described [27,28,56,57]. Orchiectomy was performed on males at 30 days just prior to reaching sexual maturity [27,28], and females underwent an ovariectomy at 2 months of age [56,57]. After surgery, all mice received adequate monitoring and medication. Mice that received a gonadectomy were compared to their age- and sex-matched littermate controls when they reached 4–5 months of age.

### 4.3. Electrophysiological Programmed Stimulations Studies (EPS)

AF inducibility was assessed using EPS, as previously described [58,59]. In brief, a Transonic (Ithaca, NY, USA) 1.9F octapolar electrophysiology catheter was inserted in the right atrium via the right jugular vein of the mouse. All procedures were performed under anesthesia (2% isoflurane), and body temperature was maintained at 37 °C with a heating pad. A Lead I surface ECG and a bipolar intracardiac ECG (iECG) were recorded simultaneously. The latter was obtained from the two most distal pairs of electrodes of the catheter. Adequate placement of the catheter in the right atrium was achieved when the main deflection of the iECG coincided with the P wave of the surface ECG. Right atrial pacing was performed by triggering a stimulus at twice the diastolic threshold. Each mouse underwent the same stimulation protocol (5 s at S1S1: 50–10 ms, 10 ms stepwise reduction) that was repeated 8 times. AF was defined as an irregular and rapid atrial rhythm with a variable ventricular rate, lasting more than 1 s, measured from the end of stimulation until the first P wave of normal sinus rhythm on the ECG.

### 4.4. Isolation of Mouse Atrial Myocytes

Isolation of single left atrial myocytes was performed using enzymatic digestion protocol, as previously described [32,60,61]. Mice were briefly heparinized (100 USP units, intraperitoneal injection) 15 min prior to sacrifice to prevent clotting. Mice were anesthetized by inhalation of isoflurane (2%) and sacrificed by cervical dislocation. The heart was rapidly removed and retrogradely perfused through the aorta on a modified Langendorff perfusion apparatus at constant flow rate (2.0 ± 0.1 mL/min) and temperature (37 ± 1 °C) with the following solutions: (1) 5 min with a HEPES-buffered Tyrode’s solution (in mM): 130 NaCl, 5.4 KCl, 1 CaCl_2_, 1 MgCl_2_, 0.33 Na_2_HPO_4_, 10 HEPES, 5.5 glucose, pH adjusted to 7.4 with NaOH; (2) 10 min with Ca^2+^-free Tyrode’s solution; (3) 25–30 min with the digestion solution (Ca^2+^-free Tyrode’s solution containing 73.7 U/mL type II collagenase (Worthington Biochemical Corporation, NJ, USA), 0.03 mM CaCl_2_, 20 mM taurine, 0.1% Bovine Serum Albumine (BSA); and 4) 3 min with Kraft-Brühe (KB) solution (in mM): 100 K^+^-glutamate, 10 K^+^-aspartate, 25 KCl, 10 KH_2_PO_4_, 2 MgSO_4_, 20 taurine, 5 creatine, 0.5 EGTA, 5 HEPES, 20 glucose, 0.1% BSA, pH adjusted to 7.2 with KOH. The left atrium was then isolated, minced, and triturated until yield of individual atrial myocytes. Freshly isolated myocytes underwent a Ca^2+^ readaptation protocol, during which Ca^2+^ was progressively reintroduced in 5-min steps at 0.06, 0.12, 0.24, 0.6 and then 1 mM Ca^2+^. The cells were then stored at 4 °C until use, typically one to six hours later. Only rod-shaped myocytes were selected for experiments.

### 4.5. Cellular Electrophysiology

Cellular electrophysiology experiments were carried out using the whole-cell voltage- and current-clamp recording techniques on freshly isolated left atrial myocytes from male and female mice using an AxoPatch 200B patch-clamp amplifier and pCLAMP 10.3 software (Molecular Devices, Sunnyvale, CA, USA). Cells were placed in a perfusion chamber of an inverted microscope.

#### 4.5.1. Action Potentials

Myocytes were perfused with 1 mM Ca^2+^ Tyrode’s solution and maintained at 37 °C to record AP. Cells were current-clamped in perforated patch-clamp configuration (130 ng/mL nystatin), using pipettes with resistances varying between 2 and 4 MΩ when filled with (in mM) 130 K^+^-aspartate, 6 NaCl, 0.4 HEPES (pH adjusted to 7.2 with KOH). AP were recorded at a frequency rate of 4 Hz, with a 2.5 ms depolarizating current of 400–700 pA. Data acquisition was performed at 20 kHz and lowpass filtered at 10 kHz. Membrane potentials were corrected by −10 mV to compensate for the liquid junction potential.

#### 4.5.2. Potassium Currents

Total K^+^ currents (I_peak_) were recorded, as previously described [26,27,28,59,62]. Atrial myocytes were briefly perfused at room temperature (RT) (20–22 °C) with Tyrode’s solution. The cells were voltage clamped in whole-cell configuration using pipettes filled with (in mM) 110 K^+^-aspartate, 20 KCl, 8 NaCl, 1 MgCl_2_, 1 CaCl_2_, 10 BAPTA, 4 K_2_-ATP and 10 HEPES (pH adjusted to 7.2 with KOH). Voltage protocol used to generate the total K^+^ currents consisted of 500 ms voltage steps ranging from −110 to +50 mV from a holding potential of −80 mV at a frequency rate of 0.1 Hz. Data acquisition was performed at 4 kHz and low-pass filtered at 1 kHz. Membrane potentials were corrected by −10 mV to compensate for the liquid junction potential. This is the only experiment carried out on mice aged 2 to 3 months.

#### 4.5.3. Sodium Currents

I_Na_ were recorded using pipettes filled with (in mM): 132.5 CsF, 5 NaCl, 5 MgATP, 10 ETGA, 5 HEPES (pH adjusted to 7.2 with CsOH) [26,59,60,61,63]. Cells were maintained at RT and perfused with a solution containing (in mM): 132.5 CsCl, 10 glucose, 1 MgCl_2_, 1 CaCl_2_, 20 HEPES, and 5 NaCl (pH adjusted to 7.4 with CsOH). Data acquisition was lowpass filtered at 2 kHz and digitized at 20 kHz. The voltage protocol used for I_Na_ was a holding potential of −90 mV and a series of 40 ms voltage steps, varying from −90 mV to +15 mV, in 5 mV increments at a frequency rate of 0.2 Hz.

### 4.6. Immunofluorescence

Immunofluorescence of Cx40 and Cx43 was performed on freshly isolated left atrial myocytes. Cells were allowed to adhere for 3 h on laminin coated coverslips, fixed for 20 min at RT with 2% paraformaldehyde (pH 7.2) in phosphate buffered saline (PBS) buffer, then blocked and permeabilized for 90 min in PBS with 2% normal donkey serum (NDS) and 0.2% Triton. Primary antibodies (Santa Cruz Goat Anti-Cx40 1:40 [5 µg/mL] CAS: sc–20466 and Invitrogen Rabbit Anti-Cx43 1:100 [2.5 µg/mL] CAS: 71–0700) were incubated overnight at 4 °C in PBS with 1% NDS and 0.05% Triton. Secondary fluorescent antibodies (Life Technologies Alexa Fluor® 488 anti-goat IgG [H + L] 1:600, CAS: A31572 and 555 anti-rabbit IgG [H + L] 1:600, CAS: A11055) and wheat germ agglutinin (WGA 1:200) were added in PBS with 1% NDS and 0.05% Triton for 1 h at RT. Coverslips were mounted on glass slides using DABCO-glycerol and sealed with nail polish. Confocal images were acquired with a Zeiss LSM 510 microscope at a 63× magnification zoom. Z-stacks were used for 3D visualization of the cells. The analysis was blinded and was performed on maximum intensity projections using ImageJ software (National Institutes of Health, NIH). Connexin lateralization was calculated as the percentage of Cx signal at the intercalated disks relative to the total Cx signal. 

### 4.7. Picrosirius Red Staining

Quantification of atrial interstitial fibrosis was performed using the Picrosirius red staining technique, as previously described [59]. The left atria were removed from the mouse hearts under anesthesia with 2% isoflurane, and fixed overnight with 10% buffered formalin at 4 °C. The atria were embedded in paraffin and cut to a thickness of 8 µm using a microtome. Sections were deparaffined using xylene and rehydrated with an ethanol-water gradient. Sections were then stained for 1 h with a saturated picric acid solution containing 0.1% Direct Red 80 (Sigma-Aldrich Corp., St. Louis, MO, USA), and washed twice with 0.5% acetic acid solution. The slides were dehydrated with a water-ethanol gradient before cover slipping them with Permount medium (Fisher Scientific, St-Laurent, Qc, Canada). Images were acquired using Qicam optic camera (Qimaging Corp., Tucson, AZ, USA) mounted on a brightfield microscope and analyzed using ImageJ software, version 1.8.0 (National Institutes of Health [NIH], Bethesda, MD, USA).

### 4.8. Quantitative Polymerase Chain Reaction (qPCR)

qPCR experiments were performed as previously described [59]. Left atria were briefly individually homogenized in Trizol Reagent, and RNA was extracted using chloroform. We did a purification using the Machery-Nagel Nucleospin RNA DNase kit to prevent contamination by genomic DNA. Reverse transcription was realized using the High-Capacity cDNA Reverse Transcription Kit (Applied Biosystems). The qPCR experiments were carried out using SYBR Select Master Mix on 5 ng of cDNA, on a Quantstudio 3 system (Applied Biosystems). Relative mRNA expression was quantified using the 2^−∆∆Ct^ method. Each sample was run in duplicate and normalized to three housekeeping genes (succinate dehydrogenase complex subunit A [*Sdha*], Peptidylprolyl Isomerase A [*Ppia*], ß2-microglobuline [*B2m*]). The primers have been validated for their specificity and efficacy.

### 4.9. Statistical Analysis

Results are presented as mean ± SEM. For data on isolated myocytes, n indicates the number of cells and N the number of mice included in the experimental groups. Statistical analysis was performed using GraphPad Prism 9.0 software (GraphPad Software, San Diego, CA, USA). Unpaired two-tailed Student’s t test or Pearson’s chi-square were used, where appropriate. *p* Values ˂ 0.05 were considered statistically significant.

## 5. Conclusions

In this study, we showed that male mice have a higher degree of atrial connexin lateralization, larger atrial myocytes, and higher atrial mass than females. Orchiectomy reduced AF susceptibility in males and abolished sex differences in connexin lateralization and myocyte dimensions, suggesting that these AF substrates are androgen-regulated. What is equally important is that these results, obtained in healthy young adult mice, indicate that sex differences in AF maintenance mechanisms are present in the absence of age-related comorbidities. Collectively, these data suggest that male atria are more prone to AF, which may contribute to the male prevalence of AF.

## Data Availability

The data that support the findings of this study are available from the corresponding author on reasonable request.
